# Enhancing healthcare cost transparency: assessing implementation challenges, criticisms, and alternative solutions

**DOI:** 10.3389/frhs.2024.1379416

**Published:** 2025-01-13

**Authors:** Sonia Ali Malik

**Affiliations:** Department of Pulmonary/Sleep Medicine, University of Utah, Salt Lake City, UT, United States

**Keywords:** cost, price transparency, CMS, benchmarks, healthcare

## Abstract

The United States healthcare system grapples with a staggering estimated waste of $935 billion, with pricing failure contributing a substantial $240.5 billion. This paper explores an innovative solution to combat rapidly escalating healthcare costs by proposing measures that would complement the mandated disclosure of healthcare prices. The Centers for Medicare and Medicaid Services (CMS) introduced a significant rule for hospital pricing, effective January 1, 2021, aiming to enhance transparency and empower patients to make value-based decisions. However, this rule has faced criticisms on various policy grounds which this examination delves into. To address these concerns and enhance the effectiveness of price transparency, this paper suggests complementary and/or alternative strategies and solutions while also examining the engagement of enrollees in price transparency tools.

## 1 Introduction

In a nation confronted by the substantial challenge of healthcare costs, clarity and transparency stand as indispensable pillars for effectively managing the intricacies of medical expenses. Despite the aspiration for an ideal where families aren't ensnared in the labyrinthine intricacies of opaque billing practices during medical emergencies, the reality in the United States paints a starkly different picture—one characterized by soaring healthcare expenditures, projected at a staggering $935 billion (1). This fiscal strain transcends mere statistical data; it manifests as a daily struggle for millions, jeopardizing their financial stability and overall well-being.

In response to this pressing dilemma, the Centers for Medicare & Medicaid Services (CMS) undertook a significant effort. Commencing on January 1, 2021, a substantial regulatory mandate was initiated—a directive compelling hospitals to disclose their standard charges, thereby illuminating the obscure terrain of healthcare pricing (2). This mandate, covering five distinct categories of negotiated charges, goes beyond bureaucratic protocol (2). It represents a moment of reform aimed at empowering patients through improved transparency, reducing costs, promoting competition, and providing individuals with the tools needed to navigate healthcare decisions effectively. The 2024 final rule from Medicare regarding hospital outpatient payments introduces revisions to the price transparency regulations. These updates require hospitals to disclose charge information using a more precise template (3).

However, amidst the urgency underscored by this initiative, lingering questions persist. How efficacious are these transparency measures in practical application? Do they authentically empower consumers, or do they risk devolving into symbolic gestures of change? The expedition into the domain of healthcare cost transparency embodies a multifaceted odyssey, characterized by inherent challenges, robust criticisms, and an unwavering pursuit of pragmatic solutions.

## 2 Literature review

Navigating the landscape of price transparency tools reveals a nuanced tapestry of research findings, highlighting both the potential and the pitfalls of these initiatives.

### 2.1 Effectiveness of price transparency tools

In a seminal study by Desai et al., the implementation of price transparency tools yielded unexpected results—no discernible reduction in expenditures was observed (4). Despite the promise of transparency, engagement rates remained tepid, with a mere 10% of employees actively seeking price estimates through online platforms. Similarly, another investigation led by Gul et al. (5) concluded that regulatory efforts had limited impact, evident from significant price disparities among hospitals. This revelation underscores the substantial challenge in aligning intentions with outcomes in the domain of healthcare cost transparency.

Conversely, the introduction of hospital price transparency tools notably reduced the cost of shoppable services. Specifically, for laboratory tests, these tools led to a 1%–4% decrease in prices (6). Furthermore, the utilization of price transparency data was associated with reduced total claims payments for common medical services. The extent of the reduction was most pronounced for advanced imaging services and least for clinical office visits, leading to the conclusion that providing patients access to pricing information before receiving clinical services may result in decreased overall payments for healthcare (7).

Delving deeper into the implications of price transparency, Chen and Miraldo’s meta-analysis unveils a nuanced landscape (8). It highlights significant variability in the effects across different countries and periods, attributing this heterogeneity to factors such as low usage rates of transparency tools, diverse implementation methods, and varying healthcare services assessed (8). Crucially, the study identifies that the effectiveness of transparency tools is heavily influenced by their design features and the presence of complementary measures like reference pricing programs. Additionally, it reveals that transparency impacts not only demand-side behaviors, such as patient choices and provider selection, but also supply-side mechanisms, including hospital competition, reputation, and strategic pricing behaviors (8). These insights suggest that for transparency tools to effectively control healthcare spending, policymakers must consider both the economic behaviors of consumers and the strategic responses of providers, tailoring interventions to specific healthcare contexts and regulatory environments.

### 2.2 Utilization and engagement

Further investigation into consumer engagement with price transparency tools reveals a diverse spectrum of behaviors and attitudes. Higgins et al.’s extensive survey, covering 31 states, illustrates modest adoption rates, with only 2% of users utilizing available price transparency tools (8). This finding is echoed by subsequent research conducted by Aetna, where a mere 3.5% of its members were found to engage with online platforms, underscoring the necessity for alternative approaches to bolster engagement (9).

The tepid utilization of price estimator tools can be attributed to various factors. Initially, hospitals’ non-compliance during the implementation phase restricts accessibility. Furthermore, the necessity for internet access acts as a significant barrier to utilization (10). Concerns have also been raised regarding the accessibility and practicality of price information for all patients, especially those with low health literacy or limited internet access (11). These insights highlight the critical need for more inclusive and user-friendly solutions to truly empower patients and make healthcare pricing transparency effective.

## 3 Policy considerations and criticisms

Amidst the discourse surrounding price transparency initiatives, critical policy considerations and criticisms emerge. Skepticism abounds regarding the potential barriers posed by pricing information to healthcare access, the logistical hurdles of disclosure, and the specter of collusion among providers (8). These challenges underscore the imperative for nuanced policy solutions that strike a delicate balance between transparency and pragmatism.

## 4 Actionable recommendations

Analytically, the literature emphasizes the imperative of proposing actionable recommendations that effectively translate intentions into tangible impacts within the domain of healthcare cost transparency. To complement CMS price transparency initiatives, it’s essential to explore both existing and novel supplementary strategies. These may encompass:

### 4.1 Integrated payments and care continuum

This model of releasing price for integrated payments and care continuum not only complements but also enhances the CMS hospital price transparency initiative by providing a comprehensive solution to healthcare cost management. Instead of billing separately for each individual service or component of care, healthcare providers receive a single, bundled payment for managing the entire care episode. This bundled payment covers all aspects of care, including diagnostic tests, consultations, treatments, procedures, medications, rehabilitation services, and any necessary follow-up care. By encompassing the full continuum of care within a single payment structure, integrated payments incentivize healthcare providers to work together seamlessly across different specialties and care settings.

The University of Utah Health’s implementation of this model illustrates its practical application (12). Their adoption of an all-inclusive cost model offers complete transparency, enabling patients to accurately estimate their out-of-pocket expenses for procedures or doctor visits. By incorporating the full scope of care costs in their estimates, patients gain a clear understanding of their financial responsibilities, contributing to trust in the healthcare system and improving the overall patient experience.

Streamlining billing, fostering collaboration among healthcare providers, and improving care coordination, this model aligns with CMS goals while ensuring comprehensive cost transparency and management. Care continuum pricing considers patients’ holistic needs from diagnosis to recovery, facilitating appropriate interventions and follow-up care.

Integrated Payments and Care Continuum Pricing offer:

#### 4.1.1 Enhanced care coordination

Aligning financial incentives and promoting collaboration streamlines care transitions and reduces fragmentation.

#### 4.1.2 Improved quality of care

Focusing on the entire care continuum encourages patient-centered care, evidence-based practices, and outcomes optimization.

#### 4.1.3 Cost containment

Bundling payments for entire care episodes reduces administrative overhead, redundant services, and unnecessary utilization, containing costs.

#### 4.1.4 Patient-centeredness

Streamlined billing and simplified financial transactions create a transparent and patient-friendly experience, prioritizing recovery over complex billing.

#### 4.1.5 Innovation and value creation

These models drive innovation in care delivery, coordination technologies, and value-based care, improving healthcare quality, efficiency, and affordability.

Overall, CMS should encourage the adoption of integrated bundled pricing models, aligning with the goals of the CMS hospital price transparency initiative. Integrated bundled pricing offers upfront visibility into total care costs for patients and fosters coordination among providers to deliver high-quality, cost-effective care. This paradigm shift emphasizes collaboration, efficiency, and patient-centeredness, complementing the CMS initiative with a holistic solution to healthcare cost management.

### 4.2 Pairing reference pricing with price transparency

Pairing Reference Pricing with price transparency involves establishing predetermined costs for healthcare services. If care aligns with or falls below the reference price, the insurer covers the entire cost, excluding deductibles and coinsurance. However, exceeding the reference price incurs additional charges. This approach aims to incentivize both consumers and providers to select and offer more cost-effective care options (13).

While not universally adopted, reference pricing is gaining recognition among insurers as a tool to promote cost-effective healthcare utilization. Some insurers have already integrated reference pricing into their benefit designs for specific procedures or services, while others are exploring or implementing such initiatives.

Insurers can seize this momentum by expanding the use of reference pricing, especially given regulatory initiatives like the CMS 2021 price transparency rule. By enhancing transparency and affordability in healthcare, insurers can empower consumers to make informed decisions and stimulate competition among providers to offer lower-cost services. For instance, Safeway grocery store successfully implemented transparency and reference pricing for procedures like colonoscopies, resulting in a 34.2% reduction in patient out-of-pocket payments and a $1.70 million decrease in employer spending (14). The overall cost for shoppable services depends on a hospital’s reference price threshold and the extent of consumer adoption of lower-cost providers.

Overall, while reference pricing for price transparency may not be universally prevalent among all insurers, it is an emerging strategy that many in the healthcare industry are considering and implementing. By leveraging reference pricing alongside transparency efforts, insurers can proactively enhance price transparency, promote cost-effective care, and improve healthcare affordability for consumers.

### 4.3 Pairing rewards programs with price transparency

The concept of combining price transparency with a rewards program originates from a study conducted by Whaley and colleagues (15). This study investigated a rewards program implemented in 2017 by twenty-nine employers, covering 269,875 eligible employees and dependents. Patients opting for shoppable services received a cash reward ranging from $25 to $500 when choosing a designated, lower-priced provider. The outcomes revealed a notable 2.1 percent relative reduction in prices across all eligible services during the first twelve months of the rewards program. The cash incentive not only facilitated a reduction in prices but also motivated individuals to actively use the pricing tool. The integration of price transparency with a rewards program aims to provide a financial incentive for individuals to seek lower-priced services. While the concept of pairing rewards programs with price transparency shows promise, its widespread implementation by insurance companies remains limited. To enhance effectiveness, insurers can collaborate with hospitals and providers to expand eligible services for rewards, improve visibility and accessibility of price transparency tools, and explore innovative incentives for cost-conscious healthcare choices.

### 4.4 Voluntary out-of-pocket cost model

As an alternative to the CMS-proposed price transparency requirement, urging for the adoption of a voluntary price information model that prioritizes furnishing patients with precise out-of-pocket costs over disclosing the total charge. Critics argue that the current disclosure of hospital price information, which represents negotiated rates, offers limited utility for patients, and may inadvertently encourage collusion among providers, potentially undermining distinctive agreements between hospitals and specific payers (10). The proposed solution suggests that insurers offer enrollees an internet-based self-service tool, providing real-time personalized access to cost-sharing information, including estimates of patient cost-sharing liability. Additionally, hospitals could offer patients an out-of-pocket cost estimator tool, utilizing an algorithm that takes into account the patient’s insurance, deductible, and co-insurance.

For instance, the University of Utah has implemented an expense estimator called “My Sure Pay Health,” offering patients pre-visit access (16). “My Sure Pay Health “ utilizes an algorithm that considers a range of factors, including the patient’s insurance plan (whether it’s Medicare, Medicaid, private payer, or self-pay), deductible, and co-insurance. Self-pay patients receive an estimate of how much their services cost, minus a 30% discount. Using this data, the tool generates personalized estimates, ensuring patients understand their financial responsibility well before their healthcare visit.

In comparison to the newly mandated government requirement by CMS, “My Sure Pay Health” offers a more personalized and real-time approach to cost estimation. Unlike the CMS requirement, which mandates all plan issuers to offer price information to insured parties, “My Sure Pay Health” tailors cost estimates based on individual insurance plans and other relevant factors and gives an all-inclusive cost. Additionally, the tool’s implementation at the University of Utah reflects a proactive stance on price transparency, potentially boosting patient satisfaction and engagement. Although new and not widely adopted, it’s considered more user-friendly than insurance website estimators.

However, to fully assess the effectiveness and usability of “My Sure Pay Health” in comparison to the CMS requirement, further research is needed. Future studies could evaluate the tool’s usage rates, accuracy of cost estimates, and impact on patient decision-making. Additionally, comparisons could be made regarding the accessibility and comprehensiveness of cost-sharing information provided by each approach.

### 4.5 Pairing price transparency tool with quality rating

Given the correlation between elevated costs and enhanced quality of care, a recommended approach involves implementing a cost estimator featuring quality ratings for medical providers. The envisaged tool should prioritize user-friendly interfaces, encompassing clear presentations of price, quality, and value to facilitate provider comparisons. It should also incorporate detailed information on the quality of care and consider patient-reported experience data. A significant hurdle in existing pricing tools is the simultaneous provision of cost and quality information to signal value to consumers. The suggested tool, which integrates price transparency with quality metrics, is proposed to address this challenge, aiding consumers in making informed decisions, particularly when opting for lower-priced alternatives.

### 4.6 Pairing preventive care with price transparency tool

The substantial impact of chronic diseases on healthcare spending, as highlighted by the CDC, reveals that nearly 75 percent of expenditures can be attributed to preventable chronic conditions (17). Recognizing the widely accepted belief that preventive care plays a pivotal role in curbing healthcare costs, this advocates for the creation of a sophisticated price transparency tool specifically tailored to support preventive care initiatives.

This envisioned tool goes beyond the conventional by actively promoting preventive care among enrollees. Upon logging in, the tool not only serves as a prompt to remind consumers about the critical importance of preventive exams but also acts as an incentive by providing reduced cost estimates for sought-after care when coupled with preventive measures. This innovative approach seeks to alleviate the economic strain imposed by chronic diseases while fostering a culture of proactive healthcare management.

### 4.7 Pairing with benchmarks

The integration of benchmarks with pricing tools is essential to empower patients in assessing the fairness of negotiated prices. Pairing price transparency tools with benchmarks, such as Medicare reimbursement rates, significantly enhances the CMS price transparency mandate by providing context and comparability for patients. While the CMS mandate requires hospitals to disclose prices, benchmarks allow patients to assess the fairness and value of these prices, facilitating more informed decision-making. This approach simplifies the complexity of comparing disparate prices and transforms raw data into actionable insights. Additionally, benchmarks encourage providers to align their prices with recognized standards, potentially leading to overall cost moderation and a better balance between cost and quality in healthcare services. This integration thus supplements the CMS mandate by not only increasing transparency but also driving more effective and value-oriented healthcare choices.

## 5 Engaging enrollees in price transparency tools

To effectively involve enrollees in price transparency tools mandated by CMS, hospitals and insurers can implement several strategies tailored to enhance awareness, accessibility, and usability. This is crucial because limited evidence from studies (2–8), suggests that hospital price transparency had an insignificant impact on consumers’ total payment due to low usage of transparency tools (9). Here are some key approaches:

1. User-Friendly Design: Enhance price transparency tools for easy navigation, side-by-side comparisons, and clear presentation.
2. In-Person Assistance: Provide help from trained social workers or staff to assist patients with tool navigation, cost breakdowns, and healthcare literacy.
3. Quality Metrics Display: Increase engagement by integrating quality metrics alongside price information, enabling patients to assess the overall value of healthcare services.
4. Cash Rewards: Implement cash reward incentives for patients who utilize price transparency tools to find lower-cost providers, potentially including waivers for copays as a financial incentive.
5. Designated Support Staff: Establish a team of designated support staff who are well-trained to address patients’ questions about price transparency tools, ensuring ongoing assistance and clarification.
6. Centralized Pre-Access Centers: Establish centralized centers for patients to verify online estimates, engage in early financial discussions, and receive real-time validation of costs.
7. Social Media Campaigns and Email Alerts: Utilize social media campaigns and email alerts to raise awareness about price transparency tools and encourage individuals to use them, highlighting their benefits and features.
8. Financial Awareness Scorecards: Introduce scorecards to help patients understand their spending patterns, fostering financial awareness and empowering them to make informed healthcare decisions.

## 6 Policy implication

The multifaceted exploration of healthcare cost transparency initiatives illuminates critical policy considerations and potential avenues for improvement. First and foremost, policymakers must acknowledge the nuanced challenges inherent in implementing transparency measures and address concerns regarding accessibility, usability, and effectiveness. The efficacy of transparency tools hinges not only on their design and implementation but also on their ability to align with consumer needs and provider practices. Integrating benchmarks, such as Medicare rates, enhances transparency, facilitating informed decision-making and cost moderation. Tailored tools promoting preventive care can alleviate the economic burden of chronic diseases. Furthermore, policymakers should consider integrating payment models that incentivize collaboration and efficiency among healthcare providers. Integrated payment models, like bundled payments for entire care episodes, streamline coordination, billing, and patient experiences. By encouraging high-quality, cost-effective care, these models have the potential to reduce healthcare costs while improving delivery efficiency. Additionally, policymakers should explore strategies to enhance consumer engagement with price transparency tools, including user-friendly design, in-person assistance, and incentives for utilization. By improving awareness, accessibility, and usability, policymakers empower consumers to make informed decisions, ultimately driving down costs and improving healthcare outcomes.

## 7 Conclusion

In conclusion, while the journey towards meaningful price transparency in healthcare is fraught with challenges, it is imperative to recognize the potential for innovation and transformation. Policymakers and stakeholders must navigate this terrain with a clear-eyed understanding of both the promise and the pitfalls, leveraging evidence-based strategies to pave the way towards a more equitable and sustainable healthcare landscape.
